# Myofilament Calcium Sensitivity: Consequences of the Effective Concentration of Troponin I

**DOI:** 10.3389/fphys.2016.00632

**Published:** 2016-12-21

**Authors:** Jalal K. Siddiqui, Svetlana B. Tikunova, Shane D. Walton, Bin Liu, Meredith Meyer, Pieter P. de Tombe, Nathan Neilson, Peter M. Kekenes-Huskey, Hussam E. Salhi, Paul M. L. Janssen, Brandon J. Biesiadecki, Jonathan P. Davis

**Affiliations:** ^1^Department of Physiology and Cell Biology and the Davis Heart and Lung Research Institute, The Ohio State UniversityColumbus, OH, USA; ^2^Cell and Molecular Physiology, Loyola University ChicagoMaywood, IL, USA; ^3^Department of Chemistry, University of KentuckyLexington, KY, USA

**Keywords:** troponin C, troponin I, effective concentration, thin filament, mathematical model

## Abstract

Control of calcium binding to and dissociation from cardiac troponin C (TnC) is essential to healthy cardiac muscle contraction/relaxation. There are numerous aberrant post-translational modifications and mutations within a plethora of contractile, and even non-contractile, proteins that appear to imbalance this delicate relationship. The direction and extent of the resulting change in calcium sensitivity is thought to drive the heart toward one type of disease or another. There are a number of molecular mechanisms that may be responsible for the altered calcium binding properties of TnC, potentially the most significant being the ability of the regulatory domain of TnC to bind the switch peptide region of TnI. Considering TnI is essentially tethered to TnC and cannot diffuse away in the absence of calcium, we suggest that the apparent calcium binding properties of TnC are highly dependent upon an “effective concentration” of TnI available to bind TnC. Based on our previous work, TnI peptide binding studies and the calcium binding properties of chimeric TnC-TnI fusion constructs, and building upon the concept of effective concentration, we have developed a mathematical model that can simulate the steady-state and kinetic calcium binding properties of a wide assortment of disease-related and post-translational protein modifications in the isolated troponin complex and reconstituted thin filament. We predict that several TnI and TnT modifications do not alter any of the intrinsic calcium or TnI binding constants of TnC, but rather alter the ability of TnC to “find” TnI in the presence of calcium. These studies demonstrate the apparent consequences of the effective TnI concentration in modulating the calcium binding properties of TnC.

## Introduction

The Ca^2+^ sensitivity of cardiac muscle contraction is compromised by many genetic and acquired cardiomyopathies (Hamdani et al., 2008; Tardiff, 2011; Liu et al., 2012a,b). This is of major significance considering it has been suggested that any chronic change in the Ca^2+^ sensitivity of cardiac muscle will eventually lead to a cardiomyopathy (Willott et al., 2010; Davis J. et al., 2016). On the other hand, we have recently demonstrated that chronic Ca^2+^ sensitization via gene therapy does not lead to disease and can be utilized to protect and therapeutically aid the heart in a murine model of myocardial infarction (Shettigar et al., 2016). Consistent with these findings, not all disease associated mutations that alter the Ca^2+^ sensitivity of cardiac muscle have complete penetrance (Tardiff, 2011; Ploski et al., 2016). Thus, either altering Ca^2+^ sensitivity does not always lead to disease or certain alterations in Ca^2+^ sensitivity are more or less easier to compensate. In any regard, with a deeper understanding of the molecular mechanism(s) that control the Ca^2+^ sensitivity of cardiac muscle, even better design strategies can be employed to correct or compensate for aberrant Ca^2+^ binding to improve cardiac function and performance (Davis J. P. et al., 2016).

Ultimately, the steady-state measurement of Ca^2+^ sensitivity is dictated by an equilibrium established by the rates of Ca^2+^ binding and release (Davis and Tikunova, 2008; Chung et al., 2016). Unfortunately, it is only in the most experimentally reduced systems that Ca^2+^ binding to TnC can be directly measured (i.e., isolated troponin C (TnC) and the Tn complex; Tikunova and Davis, 2004; Davis et al., 2007; Tikunova et al., 2010). Interestingly, the vast majority of disease-associated mutations and even several of the phosphomimetics in TnI and TnT do not have much of an impact on the Ca^2+^ binding properties of the isolated Tn complex, but do so on the thin filament (Nixon et al., 2012; Liu et al., 2012b, 2014). As the biochemical systems build and become more physiological, technical limitations necessitate having to follow other experimental outputs, which are merely transformations of the actual Ca^2+^ binding event (i.e., fluorescence, actomyosin ATPase activity, motility or force; Davis et al., 2007; Tikunova et al., 2010; Sommese et al., 2013; Meyer and Chase, 2016). In these cases, it is difficult to discern intrinsic from extrinsic influences on the apparent Ca^2+^ binding properties of TnC to even know what to “fix” or target (Davis J. P. et al., 2016).

Considering TnC has only a single regulatory Ca^2+^-binding site, it has been difficult to envision how normal or aberrant alterations in so many proteins lead to changes in the Ca^2+^ sensitivity and kinetics of this single site. It is unclear whether the aberrant Ca^2+^ sensitivity shift is caused by a direct change in the intrinsic Ca^2+^ binding properties of TnC (as would be assumed in a simple two-state switch-like mechanism), and/or merely apparently shifting the Ca^2+^ sensitivity by altering subsequent downstream events in how TnC interacts with, or is influenced by, its other regulatory subunits (such as TnI and TnT, and all their interacting proteins). Further complicating molecular insight, the disease-associated protein modifications can also impact the myocyte's innate ability to tune Ca^2+^ sensitivity via phosphorylation, so that only under certain conditions might the apparent Ca^2+^ sensitivity even appear altered (Biesiadecki et al., 2007; Messer and Marston, 2014). Depending on the system being studied, the apparent Ca^2+^ sensitivity of both the biochemical and physiological systems can vary by over an order of magnitude (Biesiadecki et al., 2014). We propose a large proportion of the variability in apparent Ca^2+^ sensitivities in these different systems is subtly controlled by Mg^2+^ competition and strongly influenced by TnI availability to TnC.

With respect to TnI availability, there are fascinating phenomena that occur at the boundaries of how we think about the mechanical and chemical world. One such behavior, effective concentration, emerges when reactants are restricted to interact in very confined spaces, as occurs when two reactants are physically tethered together (Van Valen et al., 2009). TnC and TnI can be both artificially tethered in chimeras (Tiroli et al., 2005; Pineda-Sanabria et al., 2014) and more naturally in the Tn complex (Pineda-Sanabria et al., 2014; Davis J. P. et al., 2016). Due to being tethered (Van Valen et al., 2009), TnC has the potential to experience extremely high effective concentrations of TnI to extremely low (in times when TnI mobility becomes restricted). By comparing the Ca^2+^ binding properties of TnC-TnI chimeras to that of their freed counterparts, we suggest the tethering of TnC to TnI can explain: (1) why the Tn complex has such high apparent Ca^2+^ sensitivity and slow Ca dissociation (compared to the isolated protein) that is drastically reduced and accelerated, respectively, when the Tn complex is incorporated onto the thin filament; (2) why a large proportion of Tn modifications seem to have no effect on the apparent Ca^2+^ binding properties of the isolated Tn complex, yet differences emerge when placed in the context of the thin filament; (3) that there are several different molecular mechanisms within and outside of the Tn complex that influence the intrinsic and/or apparent Ca^2+^ binding properties of TnC; and (4) at least four states of TnC are required to simulate the apparent Ca^2+^ binding properties of TnC in different experimental and diseased conditions.

## Methods

### Biochemical studies

#### Proteins utilized

The TnI_128−180_ peptide was synthesized by The Ohio Peptide, LLC (Powel, OH). We generated two TnC-TnI chimeras consisting of the N-terminal domain of human cardiac TnC (residues 1–89) with the C-terminal domain of human cardiac TnI (residues 128–211) connected by a flexible and cleavable linker containing a site for the Tobacco Etch Virus protease, which contained the sequence GGAGGENLYFQG. For the F27W chimera, the endogenous Cys residues within TnC were converted into Ser and Phe 27 was converted to Trp, resulting in the following protein sequence: MDDIYKAAVEQLTEEQKNEFKAAFDIWVLGAEDGSISTKELGKVMRMLGQNPTPEELQEMIDEVDEDGSGTVDFDEFLVMMVRSMKDDSGGAGGENLYFQGLTQKIFDLRGKFKRPTLRRVRISADAMMQALLGARAKESLDLRAHLKQVKKEDTEKENREVGDWRKNIDALSGMEGRKKKFES. For the T53C-IAANS chimera, the endogenous Cys residues within TnC were converted into Ser, Thr 53 was converted to Cys (resulting in the following protein sequence: MDDIYKAAVEQLTEEQKNEFKAAFDIFVLGAEDGSISTKELGKVMRMLGQNPCPEELQEMIDEVDEDGSGTVDFDEFLVMMVRSMKDDSGGAGGENLYFQGLTQKIFDLRGKFKRPTLRRVRISADAMMQALLGARAKESLDLRAHLKQVKKEDTEKENREVGDWRKNIDALSGMEGRKKKFES) and labeled with IAANS as previously described (Davis et al., 2007).

#### Chimera expression and purification

Pet17b vectors containing the chimeras were transformed into Rosetta 2 BL21 De3 bacteria and expressed after induction with 1 mM IPTG for 4 h. The bacteria were sonicated and the resulting solution was centrifuged at 19,000 RPM at 4°C for 30 min and the supernatant was collected. Ammonium sulfate was added at 20% saturation to remove some of the contaminating proteins. The solution was centrifuged again at 19,000 RPM at 4°C for 30 min and the supernatant was collected. Ammonium sulfate was then added to 60% saturation to precipitate the chimera. The solution was centrifuged at 19,000 RPM at 4°C for 30 min with the supernatant removed. The pellet was resuspended in 30 mL Buffer A (20 mM Tris, 2 mM EDTA, 6 M Urea, 0.5 mM DTT, at pH 8.0) and dialyzed at least four times against 1 L of the same buffer. The solution was then loaded onto an SQ-15 column equilibrated with buffer A. After an initial washing with Buffer A, a gradient was applied with 0–25% of buffer B (buffer A with 1 M NaCl). Fractions were collected and then dialyzed against 4 L of 10 mM MOPS, 150 mM KCl, at pH 7.0 at least four times.

#### Steady-state fluorescence measurements

All steady-state fluorescence measurements were performed using a Perkin-Elmer LS55 spectrofluorimeter at 15°C. Trp fluorescence was excited at 295 nm and monitored at 320 nm as microliter amounts of CaCl_2_ were added to 2 ml of titration buffer (200 mM MOPS; to prevent pH changes upon addition of Ca^2+^; 150 mM KCl, 2 mM EGTA, at pH 7.0) with constant stirring. The [Ca^2+^] free was calculated using the computer program EGCA02 developed by Robertson and Potter as previously described (Davis et al., 2007). The Ca^2+^ sensitivities were reported as a dissociation constant Kd, representing a mean of at least three separate titrations ± S.E.M. The data were fit with a logistic sigmoid function (mathematically equivalent to the Hill equation). 0.5 μM human cardiac TnC^F27W^ was titrated with Ca^2+^ in the absence or presence of up to 10 μM TnI_128−180_. The F27W chimera was also titrated with Ca^2+^ in the absence or presence of 3 mM Mg^2+^.

#### Stopped-flow fluorescent measurements

Ca^2+^ dissociation rates were characterized using an Applied Photophysics model SX.20 stopped-flow instrument with a dead time of 1.4 ms at 15°C. IAANS fluorescence was excited at 330 nm with emission monitored through a 420–470 nm band-pass interference filter (Oriel, Stratford, CT). Data traces (an average of at least five individual traces) were fit with a single exponential equation to calculate the kinetic rates. The working buffer used for the kinetic measurements was 10 mM MOPS, 150 mM KCl, at pH 7.0. Ten millimeters EGTA was utilized to remove saturating Ca^2+^ from 1 μM of the human cardiac T53C-IAANS TnC (in the absence or presence of increasing concentrations of TnI_128−180_), uncleaved 0.5 μM T53C-IAANS chimera, or cleaved T53C-IAANS chimera (in the presence or absence of increasing concentrations of TnI_128−180_). The chimera was cleaved overnight at 4°C by the addition of one part TEV protease for every five parts of chimera in 10 mM MOPS, 150 mM KCl, at pH 7.0. Since we do not complex as much EGTA with Ca^2+^ as will occur during the titration experiments, we do not need to use as much MOPS to buffer the pH. Control experiments confirmed that using buffer containing 200 mM MOPS instead of 10 mM MOPS did not affect the apparent rate of Ca^2+^ dissociation from the Tn complex following T53C-IAANS TnC fluorescence (data not shown).

### Simulations and estimations

Using Scilab, an open source numerical computational package, we solved the differential equations to obtain the time-dependent concentrations of each species given a set of rate constants and initial concentrations (described below). For the two state simulations, we plotted the TnC-Ca species as the fluorescent state and for all other simulations we plotted the TnC-Ca-TnI species as the fluorescent state. For the steady-state and transient occupancy studies, the concentration of these species were subsequently converted to a percentage of the total TnC concentration and simply overlaid onto the actual experimental data. For the Ca^2+^ dissociation and association rate studies, we normalized the change in the concentration of the species overtime and simply overlaid the simulations onto the actual experimental data.

#### Estimation of the effective concentration of TnI in the troponin complex

While the effective concentration of TnI for TnC cannot be directly measured, estimations are possible. Based on the structure of the Tn complex, it appears that the tether connecting the switch peptide of TnI to the Tn complex is along residues 134–147. If we assume a 3.4 Angstrom distance (maximal extension of the residues) and assume it to be the radius of a sphere, we obtain a total volume of 1.25 × 10^−22^ m3. By calculating the molarity of 1 TnI in this space using Avogradro's number we are able to estimate an effective concentration of ~13 mM. If we include more residues, 134–155, we estimate a lower limit of ~3 mM. This is very much in line with previous estimates of the effective concentration of a chimeric TnC-TnI protein (Pineda-Sanabria et al., 2014).

#### Steady-state Ca^2+^ binding

To simulate steady-state Ca^2+^ binding when Mg^2+^ was explicitly considered, we ran initial simulations with 1 μM TnC and 3 mM MgCl_2_ to equilibrium. This was then followed by running a loop where different levels of calcium from 0.0362 to 1000 μM were inputted and run to equilibrium. For the loop, each simulation was run to a time span of at least 0.5 s for each inputted Ca^2+^ concentration to reach equilibrium. A resulting plot of pCa vs. activated TnC ([TnC-Ca-TnI]) was developed.

#### Ca^2+^ dissociation kinetics

As with steady-state calcium binding, we initially began the simulation with 1 μM TnC and 3 mM MgCl_2_ and initially ran a simulation (at least 0.5 s time span) to determine the equilibrium concentrations of species resulting from Mg^2+^ binding. This was followed by inputting [Ca^2+^] of 200 μM. After running a simulation for at least 0.5 s, we inputted [EGTA] of 10 mM and ran a simulation. A plot of time vs. [TnC-Ca-TnI] was developed and outputted to a file.

#### Transient occupancy studies/calcium input studies

We initially began the transient occupancy simulations with 1 μM TnC, 3 mM MgCl_2_, and 600 μM EGTA. After running a simulation to equilibration, we inputted [Ca^2+^] levels of 12.5, 25, 50, and 1000 into the simulation. The simulations were normalized to the highest [Ca^2+^] level and a time vs. [TnC-Ca-TnI] plot was developed. We also performed studies without EGTA simulating the response of thin filaments to different [Ca^2+^] levels: 2.5–20 μM.

## Results

**Figure 1:**
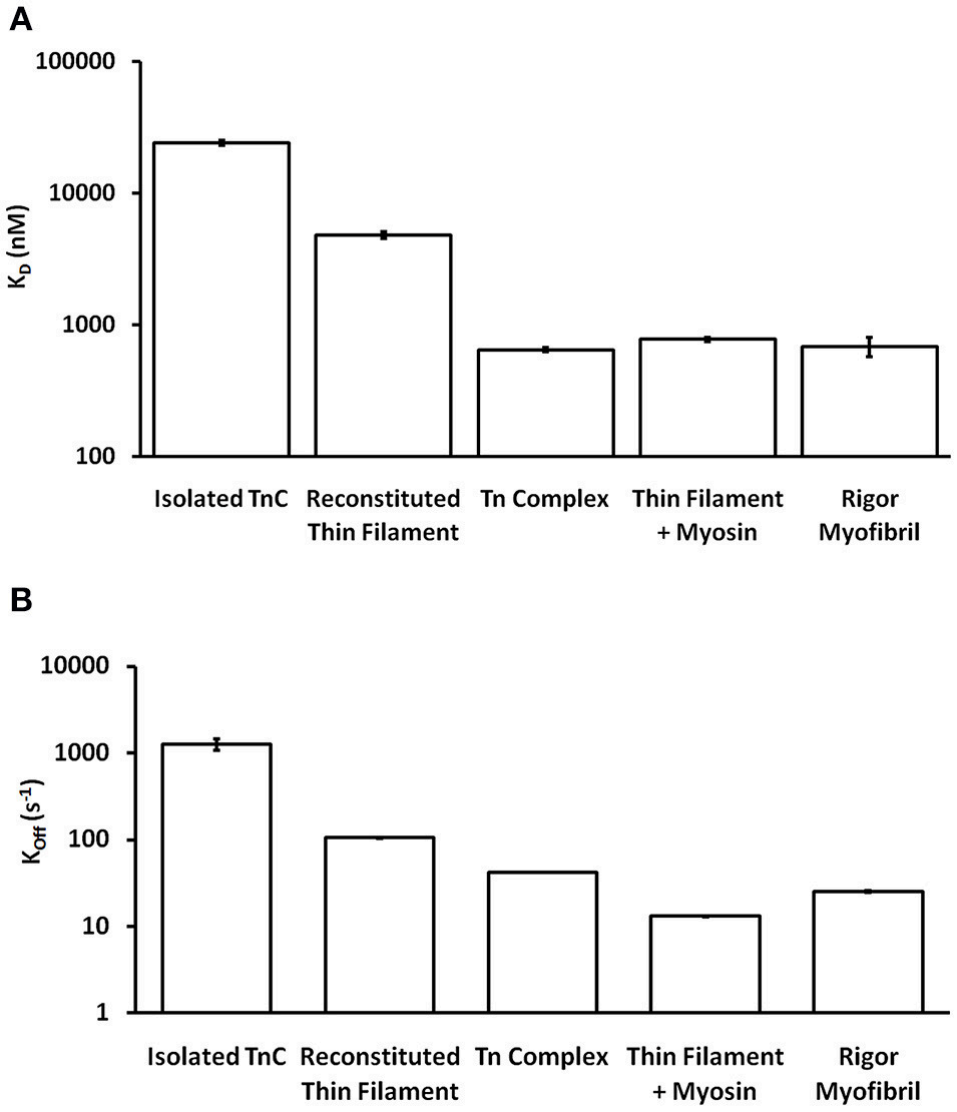
**Comparison of apparent Ca^2+^ sensitivities and dissociation kinetics of different states of TnC**. Panel **(A)** shows the apparent Ca^2+^ sensitivities ± SEM for TnC in the different biochemical systems that were extracted from our earlier publications (Tikunova and Davis, 2004; Davis et al., 2007; Little et al., 2012a,b). Panel **(B)** shows the apparent Ca^2+^ dissociation kinetics ± SEM for TnC in the different biochemical systems that were extracted from the same earlier publications. The y-axis is in a log scale making it difficult to see some of the error bars.

One of the striking and consistent findings across the literature is that the apparent Ca^2+^ binding properties of cardiac TnC vary substantially when studied in different systems (ranging from isolated TnC to muscle; Davis et al., 2007; Davis J. P. et al., 2016). Based on data from our work over the years (performed under as similar conditions as possible in simplified biochemical systems), Figure 1A demonstrates that the apparent Ca^2+^ sensitivity of TnC falls into three general Ca^2+^ sensitivity ranges. For instance, the apparent Ca^2+^ sensitivity is the lowest (highest K_d_) when only the isolated TnC is investigated, intermediate when the Tn complex is reconstituted onto the thin filament and highest in the isolated Tn complex or when the thin filament is bound by rigor myosin heads either in reconstituted thin filaments or Tn exchanged myofibrils. Similarly, the apparent rate of Ca^2+^ dissociation from TnC in these different systems somewhat scale proportionately to the change in apparent affinity (Figure 1B), giving the impression that the K_d_ changes are modulated primarily by dissociation rate changes. Thus, the same single EF-hand in the context of different systems can have drastically different apparent Ca^2+^ binding properties.

**Figure 2:**
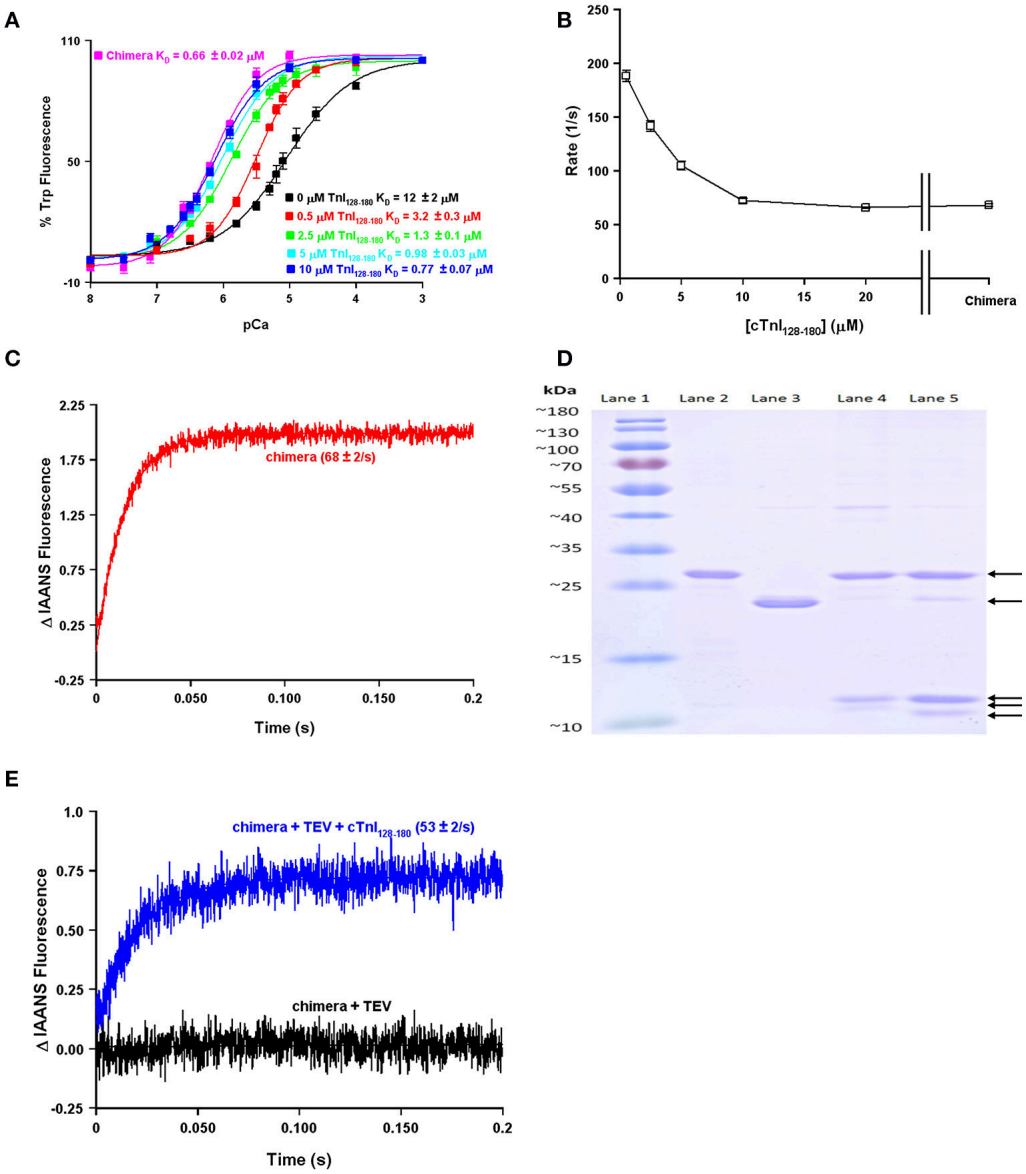
**Biochemical testing of the effective concentration of TnI**. Panel **(A)** shows the apparent Ca^2+^ sensitivities of isolated TnC^F27W^ in the absence or presence of increasing concentrations of TnI_128−180_ compared to that of the F27W chimera. Panel **(B)** shows the apparent Ca^2+^ dissociation rates from isolated TnC T53C-IAANS in the presence of increasing concentrations of TnI_128−180_ compared to that of the T53C-IAANS chimera. Panel **(C)** shows the apparent rate of Ca^2+^ dissociation from T53C-IAANS chimera. Panel **(D)** shows a representative SDS Page gel of: lane (1) molecular weight markers; lane (2) TEV; lane (3) uncut F27W chimera; lane (4) TEV digested T53C-IAANS chimera; lane (5) TEV digested F27W chimera. The arrows point to the following proteins in descending order TEV, uncut F27W chimera, TnI fragment, TnC T53C-IAANS fragment and TnC F27W fragment, respectively. The TnI fragments from the cut chimeras are of identical sequence and size, suggesting the lowest fragments are TnC. Panel **(E)** shows the apparent rate of Ca^2+^ dissociation from the TEV protease digested T53C-IAANS chimera before and after the addition of 30-fold excess TnI_128−180_.

One of the obvious differences between the simplest systems is the presence or absence of TnI. It is well-documented that the binding of the C-terminal domain of TnI to the regulatory domain of TnC increases the apparent Ca^2+^ sensitivity and slows the rate of Ca^2+^ dissociation from TnC (Figure 2; Davis and Tikunova, 2008). As can be observed in Figure 2A, increasing the concentration of TnI_128−180_ added to TnC^F27W^ increases the apparent Ca^2+^ sensitivity up to a limit. This limit approaches the apparent Ca^2+^ sensitivity of a chimeric protein in which the first 89 N-terminal residues of TnC^F27W^ were physically tethered by a short peptide linker to the C-terminal domain of human cardiac TnI (residues 128–211). Likewise, Figure 2B demonstrates that the apparent rate of Ca^2+^ dissociation from TnC (T53C-IAANS) slows with increasing concentration of TnI_128−180_down to a limit. This limit too approaches the apparent Ca^2+^ dissociation rate from a chimeric protein in which the first 89 N-terminal residues of TnC (T53C-IAANS) were physically tethered by a short peptide linker to the C-terminal domain of human cardiac TnI (residues 128−211; Figure 2C). Strikingly, the Ca^2+^ binding properties of the uncut chimeras and the two fluorescent TnCs in the presence of saturating TnI_128−180_ are similar to that of the troponin complex (compare to Figure 1). Thus, it takes over an order of magnitude more isolated TnI_128−180_ to sensitize isolated TnC to Ca^2+^ compared to the chimeras and troponin complex in which TnC and TnI are physically tethered together (high effective concentration) at a stoichiometric ratio of one to one.

Using the chimera, we can demonstrate the principle of effective concentration in yet another way. Within the peptide linker of the T53C-IAANS chimera we engineered a tobacco etch virus protease (TEV) site that can be specifically cleaved by TEV (Figure 2D). As can be observed in Figure 2D, the TEV efficiently cleaves both chimeras resulting in two bands (a TnI fragment and a TnC fragment). After TEV cleavage of the chimera the rate of Ca^2+^ dissociation is no longer observable (Figure 2E, black trace). Either the resulting rate is too fast to observe or the freed T53C-IAANS TnC N-domain's IAANS fluorescence is no longer sensitive to changes in Ca^2+^. In any regard, although we do not know whether the peptide ratios of the free N-domain of TnC to the free C-domain of TnI are at exactly one to one in the cleaved solution, a rate becomes observable again once excess TnI_128−180_ is added back to the mixture of the TEV cleaved chimera (Figure 2E, blue trace). These experiments highlight and support that there appears to be a significantly higher effective concentration of TnI within the intact chimeras and potentially the troponin complex that can drastically influence the behavior and apparent Ca^2+^ binding properties of TnC.

**Figure 3:**
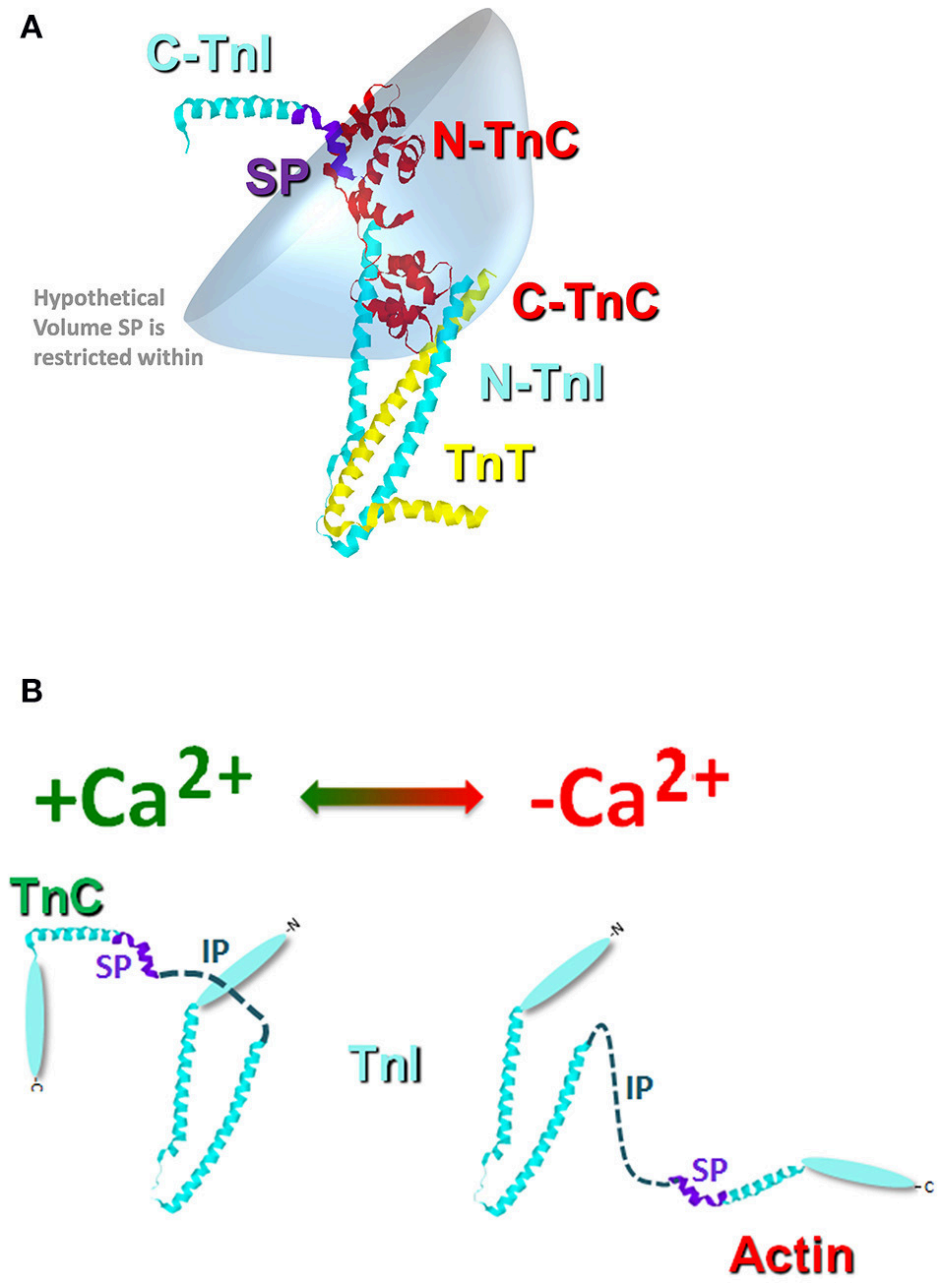
**Cartoon illustration of the effective concentration of TnI**. Panel **(A)** shows the crystal structure of the Tn complex [cyan TnI (purple switch peptide); red TnC; and yellow TnT]. The blue shaded area represents a hypothetical volume in which the TnI switch peptide may be found. Panel **(B)** illustrates the potential structural change that occurs in TnI upon Ca^2+^ removal from TnC and TnI binding to actin.

Similar to the chimeras where TnI was artificially tethered to TnC, the proper formation of the troponin complex also physically tethers TnI-TnC (Figure 3). Thus, in the troponin complex, the C-terminal domain of TnI is restricted within a small volume of space potentially orbiting (or whipping) around the N-terminal, regulatory domain of TnC (Figure 3A). If we assume this volume to be defined by a sphere with a radius equal to the length of TnI that extends from the IT arm up through the switch peptide (also assuming this stretch of amino acids to be linearly and maximally extended), the switch peptide of TnI is restricted within a maximum volume of ~1.7 × 10^6^ Å^3^. If we then place a single switch peptide into this volume we can calculate what the “effective concentration” of this peptide would be for a TnC that shares this volume space, ~3000 μM. This calculated value is similar to that estimated for another TnC-TnI chimeric protein by NMR (Hwang et al., 2014; Pineda-Sanabria et al., 2014). If we also assume that there are regions of this volume that TnC does not share, then it is possible to potentially trap, or at least temporarily restrict, TnI away from TnC, drastically plummeting the effective concentration of TnI that TnC “observes.” Such an occurrence is not difficult to imagine when the Tn complex is docked onto the thin filament, since the C-terminal domain of TnI can bind both TnC and actin (Tripet et al., 1997; Figure 3B). It may be that several proteins, TnT, Tm and myosin can influence the effective concentration of TnI that TnC observes by influencing TnI's ability to bind actin rather than by directly altering TnC's intrinsic Ca^2+^ binding properties.

**Figure 4:**
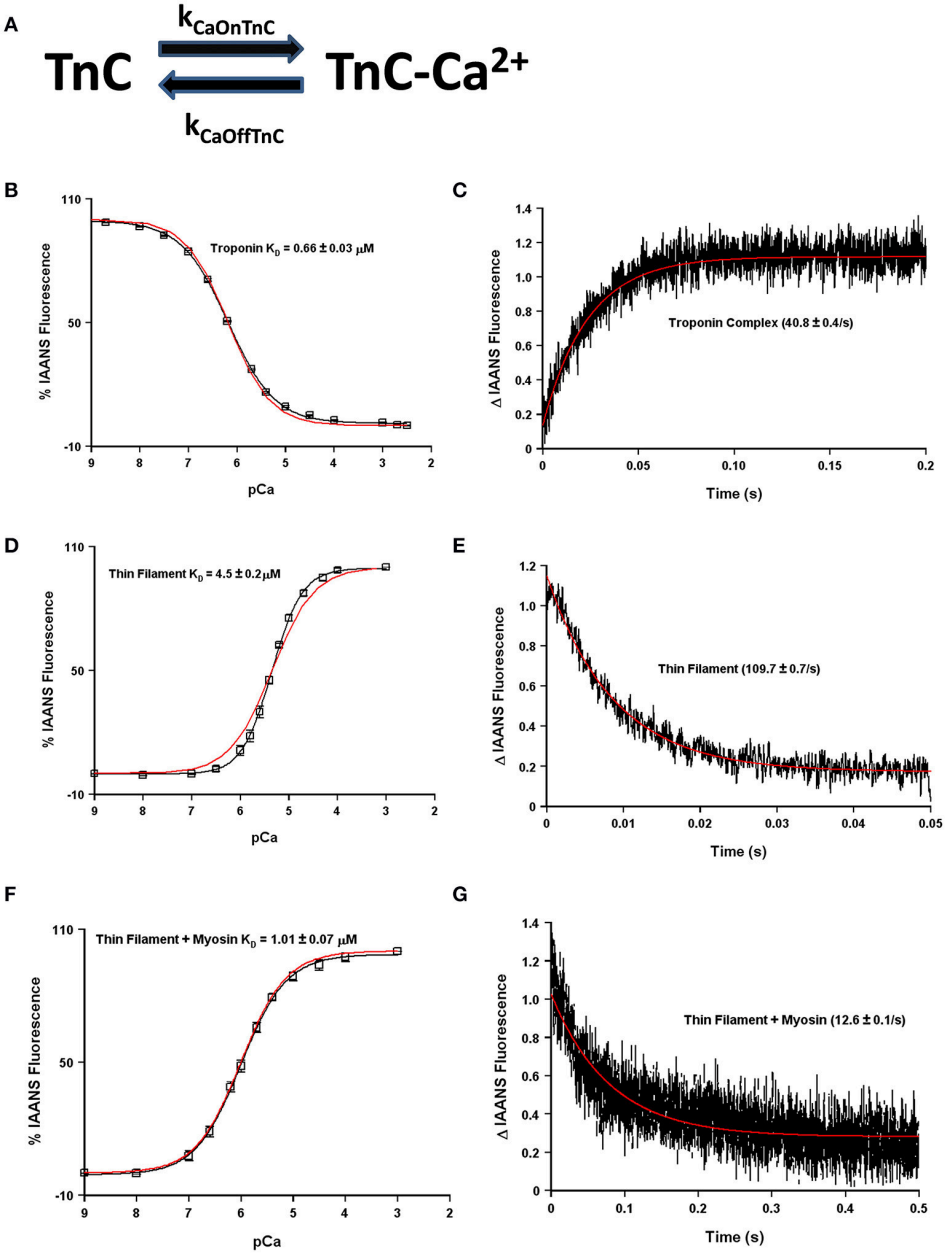
**Application of a two state model**. Panel **(A)** shows a reaction schematic for a two state model of TnC's Ca^2+^ binding. Panels **(B–G)** in black show the apparent Ca^2+^ sensitivities or dissociation rates from previously published data (Davis et al., 2007; Liu et al., 2012b), overlaid in red with a two-state model simulation. All measurements were made following the change in fluorescence of TnC T53C-IAANS in increasingly complex biochemical systems.

**Figure 5:**
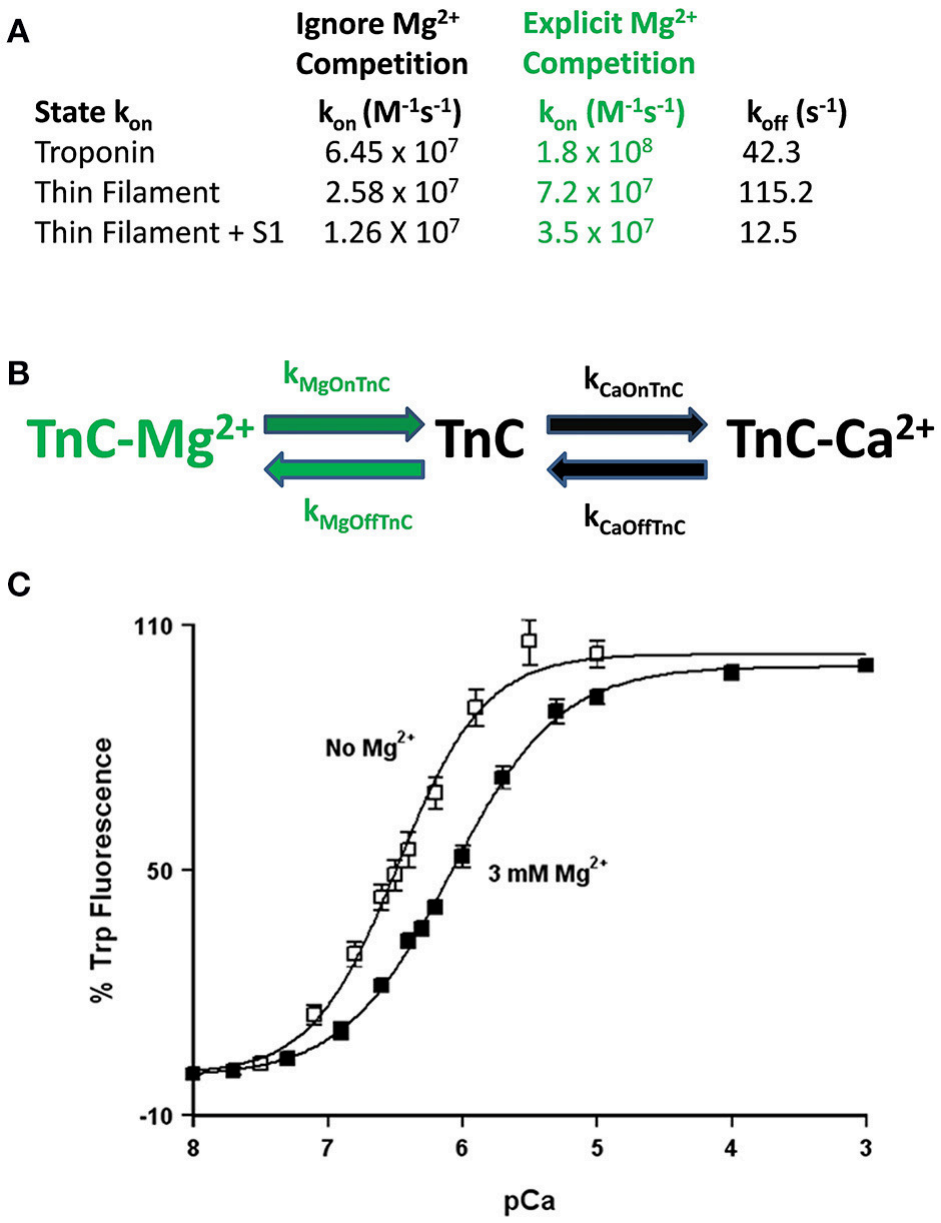
**Role of competitive Mg^2+^ binding in regulating TnC's apparent Ca^2+^ sensitivity**. Panel **(A)** shows how implicitly (Ignore Mg^2+^ Competition) or explicitly (Explicit Mg^2+^ Competition) affects the “intrinsic” Ca^2+^ association rates for TnC in different biochemical systems. Panel **(B)** shows a reaction schematic for a three state model that takes into account explicit Mg^2+^ binding to TnC. Panel **(C)** shows the apparent shift in Ca^2+^ sensitivity of the F27W chimera in the presence (solid squares) or absence (open squares) of 3 mM Mg^2+^.

The question now arises as to how many states are required to simulate, or model, the apparent Ca^2+^ binding properties of TnC and what molecular mechanisms can explain the transition between the states? Fascinatingly, a simple two-state system seems to capture quite well both the steady-state and kinetic properties of any one single system such as isolated TnC, the Tn complex, reconstituted thin filament as well as rigor myosin bound to the thin filament (Figures 4A–G). However, these simulations assume that the intrinsic Ca^2+^ association rate and dissociation rate from TnC must change in order to explain observed differences between the systems or modifications performed within a particular system (Figure 5A). A logical physical implication of this assumption is that all modifications or system changes that alter Ca^2+^ binding somehow directly influence the structure of TnC's EF-hand, Ca^2+^ coordination, or how TnC directly interacts with TnI and/or TnT. In contrast, we hypothesize that a change in the apparent Ca^2+^ binding properties of TnC can occur upon a particular perturbation without any structural change in the resulting Ca^2+^ bound structure.

This is not a trivial matter considering much effort is being put into searching for the atomic molecular mechanism(s) behind disease mutations, which are computationally forced to focus on very specific areas of interest, such as the precise coordination geometry of the Ca^2+^ ion itself (Lindert et al., 2012; Williams et al., 2016). Thus, it is critical to understand what structure or protein interactions are behind the apparent changes in Ca^2+^ binding and exchange, which may not be reflected in the Ca^2+^ bound structure itself. A prime example of this phenomenon occurs when Mg^2+^ competitively competes for Ca^2+^ binding to an EF-hand protein (Kucharski et al., 2016). Although a new structure must occur to explain Mg^2+^ binding, the Ca^2+^ bound structure will be the same regardless whether Mg^2+^ is present or not (Figure 5B). Similar to our previous data demonstrating the N-domain of cardiac TnC has a physiologically relevant and competitive Mg^2+^ affinity (Tikunova and Davis, 2004; Liang et al., 2008), the addition of Mg^2+^ to the TnC^F27W^ chimera desensitizes the apparent Ca^2+^ sensitivity of the chimera ~2.5-fold, leading to an apparent Mg^2+^ affinity of 2.1 ± 0.7 mM (Figure 5C). Since Mg^2+^ must be present in order to maintain the structural integrity of the Tn complex, we suggest Mg^2+^ competition should be taken into consideration when simulating TnC. Although subtle, only the apparent Ca^2+^ association rate utilized to approximate each system's data is dependent on whether the effects of Mg^2+^ competition are considered, not the Ca^2+^ dissociation rate (Figure 5A). In essence, Ca^2+^ cannot bind until Mg^2+^ dissociates and once Ca^2+^ binds, Ca^2+^ will dissociate at its intrinsic rate. The data in Figures 4B–F can be equally well-simulated by both a simple two state model (ignoring competitive Mg^2+^ binding) or a three state model (where TnC binds competitively Mg^2+^ or Ca^2+^; Figure 5B). However, the “intrinsic” Ca^2+^ association rate for each individual system using a simple two state model is significantly slower than what the rate must be if Mg^2+^ competition is also considered (Figure 5A). Thus, the effects of Mg^2+^ can be neglected, or lost, within a model by modulating what would appear to be the intrinsic Ca^2+^ association rate of the system.

**Figure 6:**
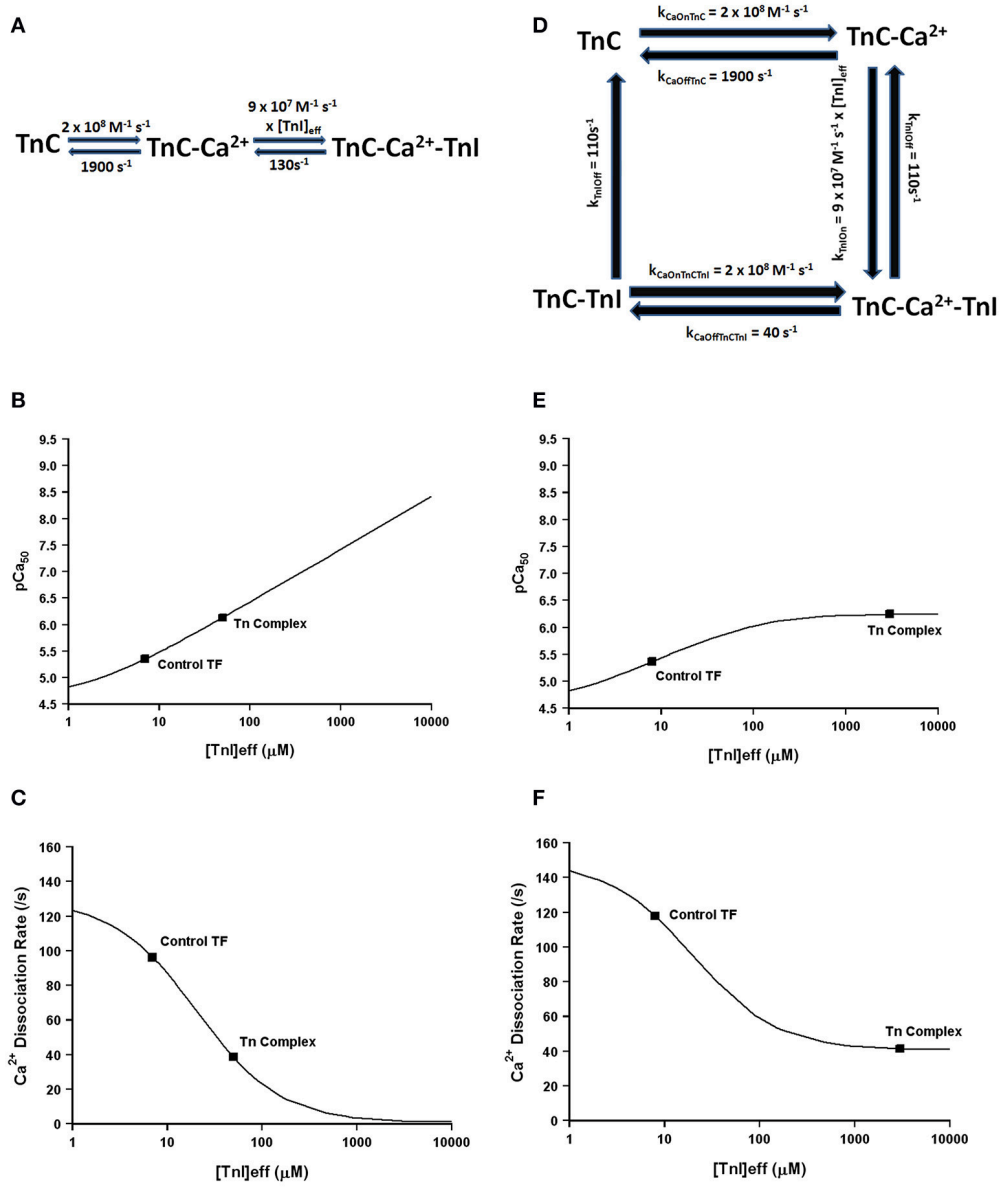
**Application of a three state model**. Panel **(A)** shows a reaction schematic for a three state model for TnC activation. Panels **(B,C)** show plots of the apparent Ca^2+^ sensitivity and dissociation kinetics derived from a three state model with increasing effective TnI concentration. Panel **(D)** shows the reaction schematic for a four state model for TnC activation. Panels **(E,F)** show plots of the apparent Ca^2+^ sensitivity and dissociation kinetics derived from a four state extended model with increasing effective TnI concentration.

Much more pronounced and generally accepted, as can be observed in Figures 1, 2, the binding of TnI to isolated TnC has a monumental impact on the apparent Ca^2+^ association and dissociation rates from TnC (Kobayashi and Solaro, 2005; Davis and Tikunova, 2008). Considering in this case two different species can bind TnC (Ca^2+^ and TnI—ignoring Mg^2+^), it is reasonable to assume that it is necessary to at least have a three state model to predict TnC behavior in muscle. In fact, a three state model has been utilized quite successfully to simulate the apparent Ca^2+^ binding properties of cardiac myofibrils (Solzin et al., 2007). Figure 6 demonstrates how a classical three state model would predict the experimental outcomes of Figures 1, 2. In a three-state model, TnC first binds Ca^2+^ with kinetics that can be described simply for the isolated TnC (either considering Mg^2+^ competition or not; Figure 6A). Although we do not know how fast TnI can actually bind Ca^2+^ bound TnC, FRET studies suggest that TnI dissociates from TnC at least as fast as ~110/s (keep in mind this rate too may be an apparent rate rather than an intrinsic rate; Ouyang et al., 2010). We estimated the TnI association rate based on a TnI affinity to TnC of 1.2 μM, which is surprisingly close to the apparent TnI_128−180_ affinity extracted from Figure 2B of ~3 μM. Figures 6B,C demonstrate a three state model incorporating TnI binding to TnC can predict the apparent Ca^2+^ sensitivities and Ca^2+^ dissociation rates of the Tn complex and thin filament by solely altering the effective concentration of TnI without having to alter any intrinsic rates of Ca^2+^ or TnI binding to/dissociation from TnC. However, unlike what was observed in Figures 2A,B, this three state model suggests that rather than there being a limit to the Ca^2+^ sensitivity and Ca^2+^ dissociation rate as the TnI concentration is increased, the pCa50 of this model continuously increases (approaching infinity), whereas the Ca^2+^ dissociation rate approaches an asymptote of zero. Thus, a three state model that does not allow for Ca^2+^ to dissociate from TnC until TnI dissociates predicts that the apparent Ca^2+^ sensitivity would approach infinity and the Ca^2+^ dissociation rate would approach zero as the TnI concentration is continually increased. Clearly this is not the case based on our peptide and chimera studies demonstrated in Figure 2. Therefore, we suggest that in addition to TnI dissociation, the TnC- Ca^2+^-TnI complex can also dissociate by releasing Ca^2+^ as well (Figure 6D). Once TnC- Ca^2+^-TnI is allowed to also dissociate via loss of Ca^2+^ as well as TnI, as the concentration of TnI is increased, the apparent Ca^2+^ sensitivity and rate of Ca^2+^ dissociation approach that of the apparent Ca^2+^ binding properties of the Tn complex (which we earlier suggested senses a very high effective concentration of TnI; Figures 6E,F).

Thus, we suggest at minimum a four state model of TnC Ca^2+^ binding and dissociation is required to accurately predict the Ca^2+^ binding properties of TnC (Figure 6D). Similar to the three state model, we first assume the initiating event is Ca^2+^ binding to the isolated regulatory domain of TnC that possesses very rapid Ca^2+^ association and dissociation rates as we have previously demonstrated to approximate the steady-state and kinetic behavior of isolated TnC. Next, TnI is allowed to bind TnC- Ca^2+^, which is governed by the product of its intrinsic on rate to TnC and the effective concentration of TnI that TnC “sees” or “senses.” Once the TnC- Ca^2+^-TnI species forms, it has two options to decay. The first pathway is via TnI dissociation, which is common to the three state model and is given a rate of 110/s consistent with FRET studies (Ouyang et al., 2010). The second pathway is via Ca^2+^ dissociation, which we have set at the rate at which Ca^2+^ has been observed to dissociate from the intact Tn complex (~40/s) with an intrinsic Ca^2+^ association rate similar to that predicted by a two-state model for the isolated Tn complex (see Figure 5A). Now we have a new state, TnC-TnI, which we assume dissociates at least as fast as TnI can dissociate from TnC- Ca^2+^-TnI complex at ~110/s. We also assume that the regulatory TnC-TnI state cannot form in the absence of Ca^2+^, consistent with experimental data (hence this reaction is not reversible).

**Figure 7:**
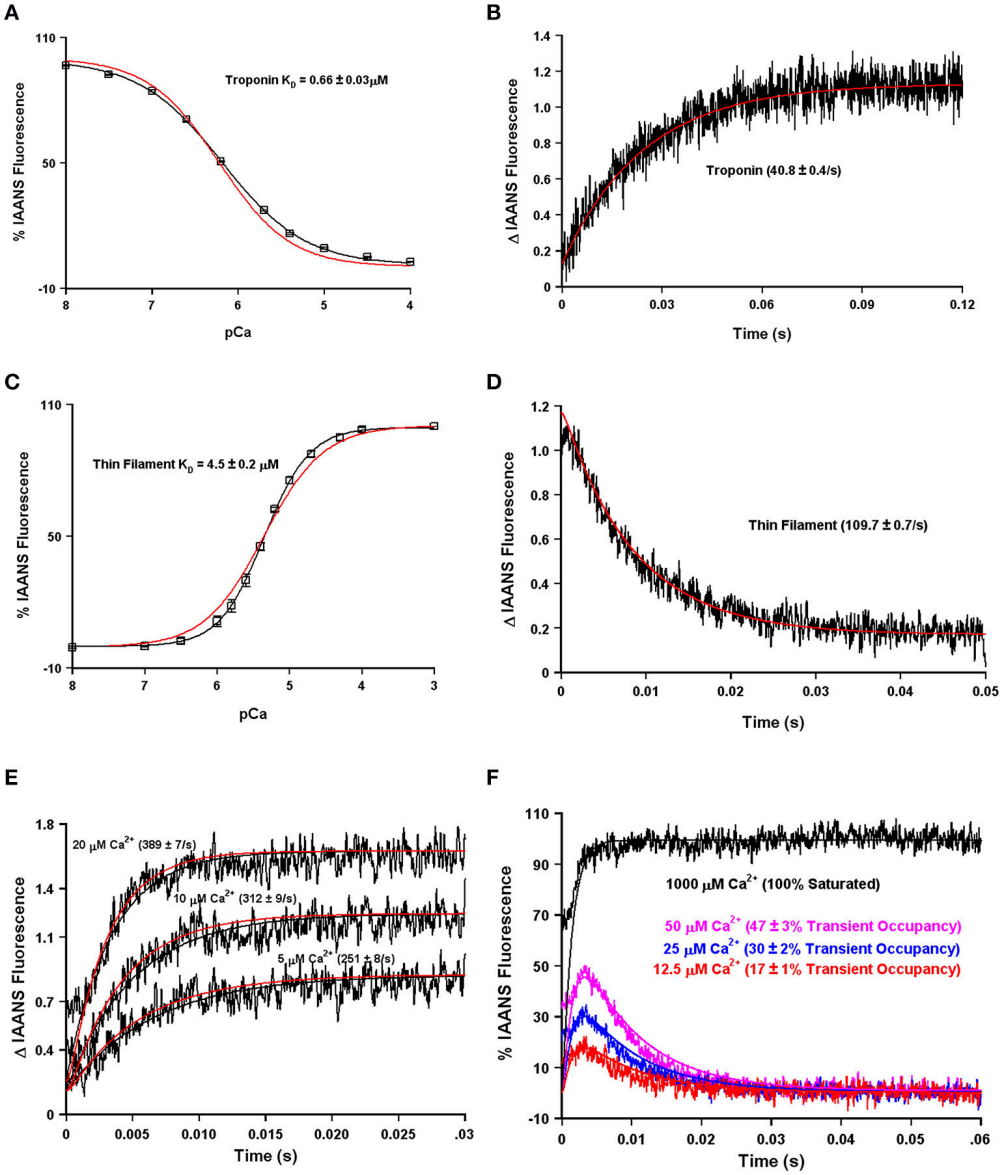
**Application of a four state model**. Panels **(A,B)** in black show the apparent Ca^2+^ sensitivity and dissociation rates from the Tn complex as previously published (Davis et al., 2007). Panels **(C,D)** in black show the apparent Ca^2+^ sensitivity and dissociation rates from the reconstituted thin filament as previously published (Davis et al., 2007). Panel **(E)** in black shows the rates of Ca^2+^ association to the reconstituted thin filaments as previously published (Liu et al., 2012b). Panel **(F)** (noisy colored curves) shows the response of the thin filaments to artificial Ca^2+^ transients as previously published (Liu et al., 2012b). For Panel **(A–E)**, the smooth red curves show the simulated output of the four state model. For Panel **(F)**, all the smooth curves represent the simulated output of the four state model.

Using our four state model and assuming a very high effective concentration of TnI (of at least 850 μM—that could actually be much higher based on the effective concentration calculations and predictions), Figures 7A,B demonstrate we can simulate the apparent Ca^2+^ binding properties of the isolated Tn complex. By only lowering the effective concentration of TnI to ~8 μM (without any intrinsic rate alterations) our four state model also predicts the apparent Ca^2+^ binding properties of the reconstituted thin filament's: (1) steady-state Ca^2+^ binding affinity (Figure 7C); (2) Ca^2+^ dissociation rate (Figure 7D); (3) Ca^2+^ association rates (Figure 7E); and (4) response to artificial Ca^2+^ transients (Figure 7F). Currently the 8 μM value for effective TnI concentration required to simulate the entire set of thin filament data is empirical. As the field learns more about the intrinsic, as well as effective concentration, of TnI that actin “sees/senses” (Figure 3B), one could begin to explicitly simulate this important interaction with TnI too.

**Figure 8:**
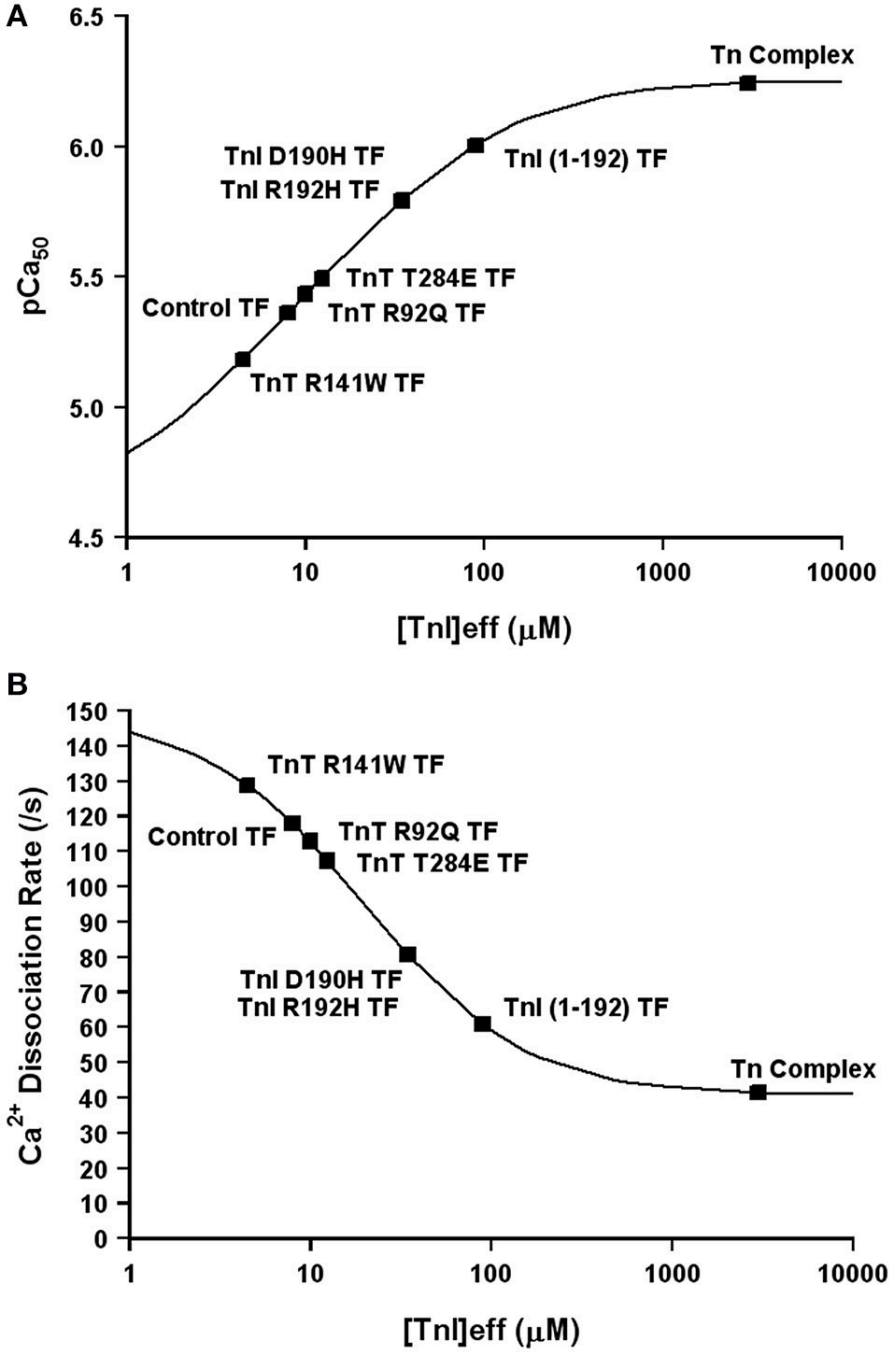
**Changes in Only the Effective Concentration of TnI Predict Ca^2+^ Binding Properties of Several TnI and TnT Modifications**. Panel **(A)** shows that the apparent Ca^2+^ sensitivities of several TnI and TnT modifications fall on the four state TnC activation curve as the effective concentration of TnI is increased. Panel **(B)** shows that the apparent Ca^2+^ dissociation rates of several TnI and TnT modifications fall on the four state TnC activation curve as the effective concentration of TnI is increased. The exact same effective TnI concentration was used for each specific protein modification in each panel.

Now that we have demonstrated that changing only the effective concentration of TnI connects TnC's Ca^2+^ binding properties between different systems, we were curious if this mechanism might also be used to explain how certain disease associated mutations alter the apparent Ca^2+^ sensitivity of TnC. Excitingly, Figures 8A,B demonstrates that nearly half of all the disease associated modifications in TnI and TnT that our laboratory has studied at both the steady-state and kinetic level can be approximated by only altering the effective concentration of TnI that TnC “sees/senses.” Thus, we suggest the ability of cardiac muscle to tune the effective concentration of TnI may be a powerful mechanism to alter the apparent Ca^2+^ binding properties of the thin filament without altering any intrinsic Ca^2+^ or TnI binding properties of TnC. However, the remaining Tn modifications we have studied require altering the intrinsic properties of TnC as well, in order to simulate the Ca^2+^ binding properties of the Tn complex and thin filament (manuscript in preparation). Collecting both steady-state and kinetic data from isolated Tn and the thin filament is essential to helping to elucidate whether a change in Ca^2+^ binding properties is due to intrinsic and/or extrinsic factors.

**Figure 9:**
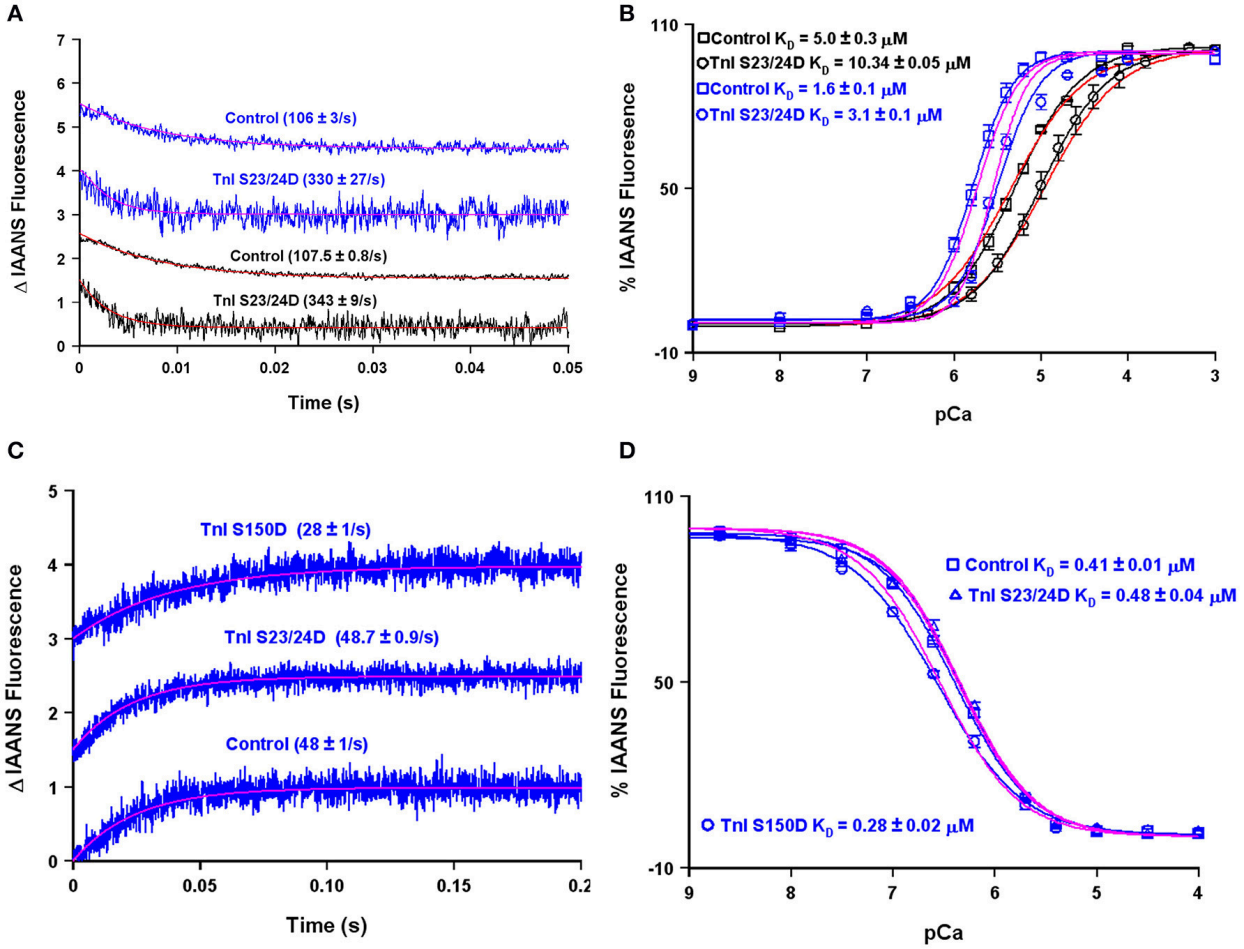
**Using the four state model to reconcile data from different research groups and predicting the molecular mechanism for S150 phosphorylation**. Panels **(A,B)** show the apparent Ca^2+^ dissociation rates and sensitivities for thin filaments containing control or S23/24D TnI, whereas panels **(C,D)** show the apparent Ca^2+^ dissociation rates and sensitivities for the Tn complex containing control, S23/24D TnI or S150D TnI. For panels **(A–D)**, the black traces are real data previously published from the Davis lab (Liu et al., 2014), the blue traces are real data previously published from the Biesiadecki lab (Nixon et al., 2014), the red traces are simulations for the Davis lab conditions, whereas the magenta traces are simulations for the Biesiadecki lab conditions.

A major modulator of thin filament Ca^2+^ binding as well as the speed of muscle mechanics is phosphorylation of cardiac TnI at serine residues 23 and 24 (Biesiadecki et al., 2014; Janssen et al., 2016). Both my, Dr. Biesiadecki's and other laboratories have independently measured the apparent Ca^2+^ binding properties of the reconstituted thin filament under similar experimental conditions using the phosphomimetic TnI in which Ser 23/24 have been mutated to Asp (Albury et al., 2012; Liu et al., 2014; Nixon et al., 2014). Not surprisingly, both of our laboratories obtain nearly identical apparent rates of Ca^2+^ dissociation from our wild type and phosphomimetic reconstituted thin filaments, suggesting our protein systems behave identically (Figure 9A). However, an apparent discrepancy arose in comparing our steady-state Ca^2+^ binding behavior of the thin filaments. Figure 9B demonstrates that my laboratory's wild type and phosphomimetic thin filaments were both substantially more desensitized to Ca^2+^ compared to the results obtained from the Biesiadecki laboratory. Unlike the kinetic measurements where saturating Ca^2+^ and EGTA are used to measure the Ca^2+^ dissociation rates and subtle differences in each concentration will have no bearing on the results, the steady-state measurements are highly dependent upon the precise concentrations of both EGTA and Ca^2+^. Excitingly, using our four state model and assuming only the concentration of EGTA was different between the laboratories (by only 10% out of 2 mM, which can easily be accounted for via differences in pipettes, equipment or H_2_O content of the EGTA powder) we are able to reconcile our experimental findings. Once we were able to correct for the Ca^2+^ buffering differences in our experimental systems, we were able to simulate the behavior of TnI S23/24D in both our data sets by making only a single intrinsic rate change of accelerating the TnI dissociation from TnC of 110–460/s (maintaining the effective concentration of TnI at 8 μM similar to the wild type condition).

Interestingly, Figures 9C,D demonstrate that unlike the reconstituted thin filament, the apparent steady-state Ca^2+^ binding properties and Ca^2+^ dissociation rate of the isolated Tn complex containing TnI S23/24D were nearly identical to that of the wild type Tn complex. This result can be simply modeled by again assuming the isolated troponin complex has a very high effective TnI concentration (>1000 μM in this case). Thus, a high effective TnI concentration can overcome the Ca^2+^ desensitizing effects of TnI S23/24D. On the other hand, TnI S150D, a phosphomimetic that models AMP kinase phosphorylation of TnI, sensitizes both the Tn complex and thin filaments to Ca^2+^, as well as slows both systems' Ca^2+^ dissociation rates (Figures 9C,D; Nixon et al., 2012, 2014). In order to simulate these results we have assumed two alterations: (1) the rate of Ca^2+^ dissociation from the TnC- Ca^2+^-TnI complex is slowed by 40% (setting a new limiting value as the effective TnI concentration is elevated) and (2) the effective concentration of TnI for the reconstituted thin filament was raised from 8 to 25 μM. Thus, our model predicts that unlike TnI S23/24D, TnI S150D increases the effective concentration of TnI that TnC “sees” when incorporated onto the thin filament. One possibility for this observation and a rationale for having to increase the effective concentration of TnI for TnC on the thin filament could be a weakened binding affinity of TnI S150D for actin. Consistent with this prediction, Salhi et al. has demonstrated TnI S150D has a weaker affinity for actin, compared to TnI S23/24D, which had a similar actin affinity as compared to wild type TnI (Salhi et al., 2016). This observation suggests mechanisms that alter the ability of TnI to interact with actin can have a profound effect of modulating the apparent Ca^2+^ binding properties of TnC potentially through the effective concentration of TnI that TnC “sees/senses.”

## Discussion

There has been great interest in developing mathematical models that can recapitulate the electrical, Ca^2+^ and mechanical responses of the heart. Although there are very good models for elements of each of these processes, no model has been able to unify all the essential steps of contraction/relaxation into a cohesive predictive or diagnostic tool. We argue that one of the problems arises in the need to oversimplify the systems, of which Ca^2+^ binding to TnC is a prime example. Based on a wealth of biochemical and physiological data it is clear that the apparent Ca^2+^ binding properties of TnC can influence both the extent and speed of cardiac muscle contraction, relaxation, and power (McDonald and Herron, 2002; Biesiadecki et al., 2014; Davis J. P. et al., 2016). By more fully understanding the underlying mechanisms that control the intrinsic and apparent Ca^2+^ binding properties of cardiac muscle it may be possible to engineer the response of the thin filament to Ca^2+^ to eventually treat various cardiovascular diseases, as we have been attempting to achieve (Davis J. P. et al., 2016; Shettigar et al., 2016).

Although each individual state of TnC (isolated TnC, Tn complex, thin filament, thin filament bound by strong cross-bridges, myofibrils, etc.) can be simulated with a simple two state system (see Figure 4), by no means does this suggest Ca^2+^ binding to TnC in muscle is simple. We argue that TnC transitions through multiple states even during a single heartbeat, which can then influence the mechanics of that heartbeat when the thin filament is altered naturally by phosphorylation or even disease. Thus, we have set out to try and understand what might be the major influences on Ca^2+^ binding to TnC as it transitions from state to state. Clearly the ability of TnC to bind TnI is one major factor that influences TnC's Ca^2+^ binding properties (Kobayashi and Solaro, 2005; Biesiadecki et al., 2014). However, even this step in the process may be more complicated than just understanding the affinity or intrinsic rate of TnI association and dissociation from TnC. We suggest there are mechanisms beyond the overall “affinity” of TnC for TnI that can feed back on TnC without altering any intrinsic parameters of TnC, such as the effective concentration of TnI that TnC “sees/senses.”

The concept of effective concentration is not a new idea (Van Valen et al., 2009), even as it applies to TnC (Hwang et al., 2014), although we are the first to try and apply the concept to mathematically describing state transitions of TnC. Based on our initial studies we speculate that nearly half of all the disease associated modifications of Tn that we have studied can be explained solely by altering the effective concentration of TnI that TnC “sees.” Other Tn modifications such as TnI S23/24D may have no effect on TnI's effective concentration, whereas others such as TnI S150D require modulating both intrinsic TnC properties as well as the effective concentration of TnI (Salhi et al., 2016). As we work through trying to model all our biochemical data, we are finding there are many ways to alter the apparent Ca^2+^ binding properties of TnC. Not all of the mechanisms are as straightforward as simply accelerating or slowing the intrinsic Ca^2+^ binding properties of TnC. In most cases, the potential mechanism behind a Tn modification is not evident until the protein modification is studied in multiple biochemical systems (at minimum the troponin complex and the reconstituted thin filament) both in the steady-state and kinetically. It is clear that Ca^2+^ triggers extensive dynamic changes in Tn that can be altered by disease associated mutations (Kowlessur and Tobacman, 2012; Liu et al., 2012b). As the model is developed to move from Ca^2+^ binding to force, we will need to also consider cooperative unit communications (Gordon et al., 2000). As one moves beyond this TnC centric view, one will also need to consider tropomyosin positions as well as TnT behavior when determining the ability of Ca^2+^ to modulate myosin binding to actin (Mijailovich et al., 2012).

We suggest the tethering of TnC to TnI and our model can help to explain: **(1)** why the Tn complex has such high apparent Ca^2+^ sensitivity and slow Ca^2+^ dissociation (compared to the isolated protein) that is drastically reduced and accelerated respectively, when the Tn complex is incorporated onto the thin filament. Figure 2 clearly shows that the apparent Ca^2+^ sensitivity and dissociation rate of isolated TnC is modulated by the concentration of TnI. Furthermore, at saturating TnI concentrations, both the apparent Ca^2+^ sensitivity and dissociation rate are very similar to the intact Tn complex as well as the chimeras. Figure 3 illustrates that the proximal confinement of TnI to TnC results in a high effective TnI concentration in the Tn complex. Figures 6, 7 use our model to show that at a high effective TnI concentration, we can accurately simulate Tn behavior and by solely lowering the effective TnI concentration we can simulate thin filament behavior. **(2)** Why a large proportion of Tn modifications seem to have no effect on the apparent Ca^2+^ binding properties of the isolated Tn complex, yet differences emerge when placed in the context of the thin filament. Figure 8 shows that the apparent Ca^2+^ binding properties of several Tn modifications can be modeled solely by changing the effective concentration of TnI on the thin filament. Furthermore, at a saturating TnI concentration, our model predicts for each of these Tn modifications that the apparent Ca^2+^ binding properties of the Tn complex are identical to that of the wild type Tn complex. Figure 9 shows an example of a modification that requires changing an intrinsic rate parameter in order to model thin filament behavior. However, raising the effective concentration of TnI to saturating levels restores the Tn like behavior. In this case, an accelerated TnI dissociation rate from TnC will be over-powered by the high effective TnI concentration. **(3)** There are several different molecular mechanisms within and outside of the Tn complex that influence the intrinsic and/or apparent Ca^2+^ binding properties of TnC. Our study focused on how altering the effective concentration of TnI can regulate the apparent Ca^2+^ binding properties of TnC. As pointed out in the manuscript and our model, we do not exclude that there are mutations/modifications in the Tn complex (or a plethora of additional proteins) that can alter the apparent Ca^2+^ binding properties of TnC though alternate mechanisms, such as altering the intrinsic rates of Ca^2+^, Mg^2+^, and/or TnI binding/dissociation from TnC. In fact, this is the point of a companion paper to be published in this thematic issue by the Biesiadecki lab (Salhi et al., 2016). Thus, all the intrinsic binding and dissociation rates (Ca^2+^, Mg^2+^, and TnI) set by the compilation of proteins in the thin and thick filaments, as well as the effective concentration of TnI, work together to set the overall Ca^2+^ sensitivity of TnC. **(4)** At least four states of TnC are required to simulate the apparent Ca^2+^ binding properties of TnC in different experimental and diseased conditions. Figures 6–9 demonstrate at minimum a four-state model is needed to successfully simulate not only the steady-state, but also kinetic behavior of a wide assortment of Tn modifications.

In conclusion, we have generated a strikingly powerful mathematical model that can simulate several different states of TnC in the presence of different Tn modifications. We have utilized the model to make predictions regarding protein interactions that fall outside of direct TnC interactions, such as TnI-actin binding (Salhi et al., 2016). Currently all that is needed to connect and simulate our data is a four state model to recapitulate TnC's apparent Ca^2+^ binding properties as well as evoking the concept of effective concentration. Considering nearly all of the myofilament protein-protein interactions that influence muscle contraction occur within confined and restricted spaces, effective concentration concepts may need to be invoked to simulate several other key reaction steps as well, especially myosin with actin (Fuchs and Smith, 2001).

## Author contributions

Performed experiments: JS, ST, SW, MM, NN, HS, BL. Designed Study: JS, ST, Pd, BB, JD. Analyzed Data: JS, ST, PJ, PK, JD.

## Funding

Supported by NIH Grants HL091986 (JD), AG051913 (JD), HL117034 (ST), HL113084 (PJ), HL1114940 (BB), HL62426 (Pd).

### Conflict of interest statement

The authors declare that the research was conducted in the absence of any commercial or financial relationships that could be construed as a potential conflict of interest.
